# Retinal Artery Occlusion in a Young Healthy Male

**DOI:** 10.51894/001c.144638

**Published:** 2025-09-30

**Authors:** Alexandra Schulte

**Affiliations:** 1 Department of Ophthalmology Henry Ford Warren

## 119

### INTRODUCTION

Vascular supply to the eye originates from the internal carotid artery, which then gives rise to the ophthalmic artery, and bifurcates into the central retinal artery and ciliary arteries. Occlusions in either of these two structures places the globe at risk for ischemic processes, and is considered an ophthalmic emergency. Central retinal artery occlusion (CRAO) and ophthalmic artery occlusion (OAO) are more often seen in older males with vasculopathic or coagulopathic history. Stroke pathway initiation is critical, however there is no consensus regarding optimal treatment, and visual prognosis is poor. Here we present a unique case of an otherwise healthy young male who presents with complete vision loss of his right eye consistent with retinal artery occlusion.

### CASE

29 y/o M presented with complete painless vision loss of his right eye (OD). Visual acuity was measured at light perception only. Pupil exam was significant for anisocoria at 6 mm OD and 5 mm left (OS) in light conditions with a 3+ afferent pupillary defect OD. Dilated retinal examination demonstrated diffuse retinal whitening in the posterior pole with a “cherry red spot”. He was diagnosed with CRAO and a full stroke workup was completed which was unremarkable. Tenecteplase (TNK) was administered. An extensive inflammatory work-up was conducted which revealed a positive ANA. Additional outpatient workup included optic coherence tomography (OCT) and fluorescein angiography (FA), which revealed diffuse macular edema, and delayed retinal and choroidal filling respectively. Collectively these findings support the diagnosis of OAO.

### DISCUSSION/CONCLUSION

Our patient’s clinical examination was evident for retinal artery occlusion, yet FA demonstrated delayed filling in both anterior and posterior chorioretinal vasculature, concerning for a more proximal blockage at the ophthalmic artery rather than the central retinal artery alone. While older vasculopathic males are most often encountered in this disease process, here we share a case of a healthy young male with otherwise negative stroke workup. A family history positive for autoimmune conditions and newfound positive ANA value should not be ignored as severe vasculitis events may present similarly. Assessing risk factors and controlling systemic disease is critical to protect the fellow eye.

